# Impact of Myopia Control Spectacle Lenses on Visual Functions in Young Adults: A Comprehensive Evaluation

**DOI:** 10.3390/jcm15093362

**Published:** 2026-04-28

**Authors:** Muteb K. Alanazi, Mohammed Alhazmi, Wafa Alotaibi, Basal H. Altoaimi, Alla I. Alshetwi, Niran A. Alanazi, Meshal D. Alotaibi, Bader S. Alqahtani, Rayan H. Almalki, Maria Liu

**Affiliations:** 1 Optometry Department, College of Applied Medical Sciences, King Saud University, Riyadh 11362, Saudi Arabia; malhazmyi@ksu.edu.sa (M.A.); walotaibi@ksu.edu.sa (W.A.); baltoaimi@ksu.edu.sa (B.H.A.); 442106147@student.ksu.edu.sa (A.I.A.); 442201756@student.ksu.edu.sa (N.A.A.); 442100452@student.ksu.edu.sa (M.D.A.); 442100190@student.ksu.edu.sa (B.S.A.); 442100381@student.ksu.edu.sa (R.H.A.); 2 School of Optometry, University of California, Berkeley, CA 94720, USA; marialiu@berkeley.edu

**Keywords:** myopia control, peripheral defocus, visual acuity, contrast sensitivity, visual field, video game performance

## Abstract

**Purpose:** To evaluate the impact of a myopia control (MC) spectacle lens incorporating peripheral defocus on functional visual parameters compared to conventional single vision (SV) lenses. **Methods:** Thirty-nine young adults (age 21.1 ± 1.3 years; spherical equivalent −2.85 ± 2.8 D) participated in this single-session, within-subject crossover design. Distance and near LogMAR VA were assessed centrally and at 22° nasal and temporal off-axis. Distance contrast sensitivity (CS) was measured across five spatial frequencies (1.5–18 cpd) and the Area Under the Log CS Function (AULCSF) calculated. Retinal sensitivity was evaluated using automated static perimetry. Dynamic visual performance was assessed using a standardized video game platform. Outcomes were compared using repeated measures ANOVA or Wilcoxon signed-rank tests. **Results:** Central VA was comparable between lens types. MC lenses significantly reduced off-axis distance VA (nasal: 0.35 ± 0.15 LogMAR; temporal: 0.20 ± 0.14 LogMAR; both *p* < 0.001) and near VA (nasal: 0.72 ± 0.25 LogMAR; temporal: 0.34 ± 0.2 LogMAR; both *p* < 0.001). Off-axis AULCSF was significantly reduced with MC lenses (nasal: 12.07 ± 5.06 vs. 26.37 ± 5.00 units; temporal: 13.00 ± 6.93 vs. 27.14 ± 4.64 units; both *p* < 0.001), while central AULCSF remained similar between lens types (SV: 27.87 ± 5.04 vs. MC: 27.15 ± 5.02 units; *p* = 0.277). No significant differences were found for visual field indices (all *p* > 0.05). Video game accuracy was comparable between lenses, but task completion time was slower with MC lenses (20.71 ± 10.08 vs. 18.39 ± 6.65 s; *p* = 0.012). **Conclusions:** MC spectacle lenses preserve central VA, CS, and visual field sensitivity but induce significant off-axis VA and off-axis CS reductions. Dynamic visuomotor accuracy is maintained, though task completion speed is modestly reduced. These functional trade-offs should be considered when prescribing MC lenses.

## 1. Introduction

Myopia, or nearsightedness, is a refractive error characterized by a mismatch between the optical power and axial length of the eye, causing parallel rays of light to focus anterior to the retina, resulting in blurred distance vision. Clinically, it is defined as a spherical equivalent refractive error of ≤−0.50 diopters (D), with high myopia (≤−6.00 D) constituting a significant subpopulation at elevated risk for vision-threatening pathology [1]. The condition has reached epidemic proportions, with global prevalence projected to rise from 1.4 billion in 2000 to nearly 5 billion by 2050, of whom approximately 1 billion are expected to have high myopia [2]. This surge, occurring over mere decades, underscores a powerful environmental influence superimposed on a well-established genetic predisposition [3].

The primary public health concern transcends the need for optical correction. Excessive axial elongation, the hallmark of progressive myopia, is the principal biometric risk factor for sight-threatening complications. These include myopic maculopathy, retinal detachment, primary open-angle glaucoma, and early-onset cataract, with risk increasing monotonically with axial length [4,5]. For instance, each 1 mm increase in axial length elevates the risk of myopic maculopathy by approximately 67% [6]. This established link between axial elongation and irreversible ocular disease has shifted the clinical paradigm from passive correction to active intervention aimed to slow axial growth.

The underlying mechanisms driving axial elongation involve complex ocular signaling pathways influenced by visual experience. Key theories implicate both central and peripheral retinal defocus. Peripheral hyperopic defocus, commonly induced by traditional single-vision spectacle lenses, is a potent stimulator for axial elongation in animal models and is believed to contribute to progression in humans [7]. Conversely, the introduction of sustained myopic defocus, particularly in the retinal periphery, has been shown to inhibit eye growth [8]. Furthermore, retinal contrast processing is implicated; specific spatial frequencies and luminance conditions, such as those prevalent in prolonged near-work (e.g., high-contrast black text on white background), may promote pro-myopic signaling through biased activation of retinal ON and OFF pathways [9].

In response, several evidence-based myopia control interventions have been developed. Low-dose atropine (0.01%) demonstrates efficacy in slowing progression, though side effects like photophobia and concerns about rebound exist [10]. Orthokeratology (ortho-k) and soft multifocal contact lenses leverage peripheral myopic defocus, showing 40–60% reduction in axial elongation compared to controls [11]. Most recently, specially designed spectacle lenses have emerged as a non-invasive, widely accessible option. Lenses incorporating Defocus Incorporated Multiple Segments (DIMS) or Highly Aspherical Lenslet Target (H.A.L.T.) technologies create a treatment zone of simultaneous myopic defocus surrounding a clear central zone. A pivotal 3-year randomized controlled trial demonstrated that DIMS spectacles reduced myopia progression by 52% and axial elongation by 62% compared to single-vision lenses [12].

Despite growing evidence supporting the efficacy of MC spectacle lens designs in slowing myopia progression, a critical knowledge gap persists regarding their impact on functional vision. The optical design principles shared across this class of lenses—particularly the simultaneous presence of a clear central zone and a peripheral defocus treatment gradient—may theoretically affect visual functions beyond high-contrast visual acuity, yet this has not been comprehensively characterized for concentric radially symmetric designs. Potential impacts on contrast sensitivity, performance in dynamic visual tasks (e.g., gaming), and peripheral retinal sensitivity remain inadequately characterized [13]. Given that these interventions are prescribed for children and adolescents during critical periods of academic and social development, any compromise in functional vision could affect performance, comfort, and long-term compliance. Therefore, a comprehensive assessment is imperative. This study aims to systematically characterize the functional visual trade-offs associated with a concentric peripheral defocus MC spectacle lens design.

## 2. Materials and Methods

This was a single-session, within-subject crossover study, conducted between February 2025 to May 2025 performed in optometry clinics at King Saud University, Riyadh, Saudi Arabia. The study was approved by the Institutional Review Board at the College of Medicine, King Saud University (Project No. E-24-9149). Written informed was consent obtained from all participants after explaining the aim and nature of the study.

### 2.1. Participants

Thirty-nine healthy young adults (8 emmetropic, 24 myopic (SE ≤ −0.50 to >−6.00 diopter), and 7 high myopic (SE ≤ −6.00 diopter) aged between 19–24 years old were recruited. All participants had astigmatism less than 2 diopters with no a history of any ocular or systemic diseases, such as diabetes, hypertension, previous ocular surgery, diabetic retinopathy, corneal opacity, cataracts, pathological myopia, high astigmatism, or glaucoma.

All participants signed an informed consent form prior their enrolment. Following this, a comprehensive examination conducted including monocular and binocular visual acuity and subjective refraction. Subjective refraction was performed to determine individualized prescriptions for both myopia control (MC) and single vision (SV) spectacles. Accommodation amplitude and facility assessed using push-up test and +2.00D/−2.00D. Interpupillary distance were measured both monocularly and binocularly. Extra-ocular muscle function evaluated monocularly to ensure normal ocular motility. A confrontation visual field screening performed to detect any gross visual field defects before formal testing. Additionally, a slit-lamp examination used to assess the anterior segment health. Pentacam AXL (OCULUS Optikgeräte GmbH, Wetzlar, Germany) was used to assess the corneal curvature, as well as to obtain axial length measurements.

### 2.2. Myopia Control Spectacle Lens Design

**Figure 1:**
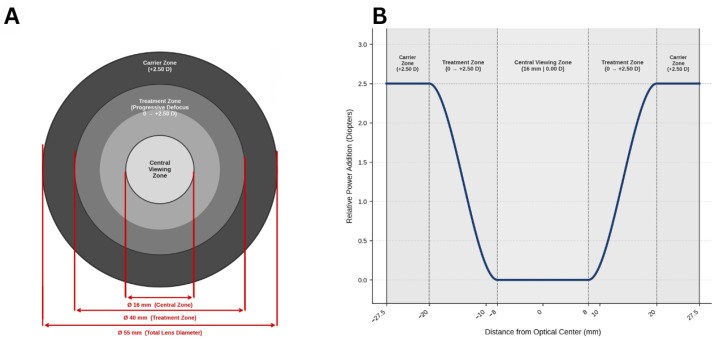
Schematic and power profile of the MYOpis Classic myopia control spectacle lens (Divel Italia, Bologna, Italy). (**A**) Concentric zone layout: central viewing zone (Ø 16 mm), treatment zone (Ø 40 mm, progressive 0 to +2.50 D), and carrier zone (Ø 55 mm, +2.50 D). (**B**) Relative power addition across the horizontal meridian showing a flat central zone (0.00 D), progressive increase through the treatment zone, and a flat carrier zone at the maximum addition of +2.50 D.

The myopia control spectacle lens evaluated in this study was the MYOpis Classic (Divel Italia, Calderara di Reno, Bologna, Italy), a commercially available concentric, radially symmetric design intended for the management of myopic progression. The lens consists of three distinct zones. The central viewing zone measures 16 mm in diameter and provides the full optical distance correction for primary gaze, ensuring uncompromised central visual acuity. This is encircled by a treatment zone extending to 40 mm in diameter, incorporating a gradual and progressive increase in plus power relative to the central zone, reaching a maximum peripheral add power of +2.50 D at the treatment zone boundary. Beyond this, a peripheral carrier zone maintains the maximum addition of +2.50 D to the full lens diameter of 55 mm. The lens power profile across the full diameter demonstrates a controlled progressive increase in peripheral power relative to the center, forming a continuous radially symmetric power gradient (Figure 1). According to the manufacturer, the maximum add power achievable at the lens edge is +2.50 D, with the precise power profile calculated based on each participant’s individual prescription and the useful lens diameter.

Prior to dispensing, all fabricated lenses were edged and mounted into a standardized unified plastic spectacle frame (lens dimensions: 54 mm width, 40 mm height). The frame was positioned on each participant such that the lenses were optically centered relative to the participant’s monocular and binocular interpupillary distance and a vertex distance of 12 mm was maintained for all participants. Centration accuracy was verified using a Topcon CL-300 Auto Lensometer (Topcon Corporation, Tokyo, Japan), confirming both the back-vertex power of each lens against the participant’s individualized prescription and the accurate alignment of the optical center. The same frame and fitting position were used consistently across both lens conditions for each participant to ensure comparable optical geometry throughout testing.

### 2.3. Visual Acuity and Contrast Sensitivity Measurements

Distance and near visual acuity of right eyes were assessed under photopic illumination conditions (426 lux) in three gaze positions: central on-axis, 22° nasal off-axis, and 22° temporal off-axis. The 22° off-axis gaze angle was selected based on optical geometry calculations confirming that this eccentricity places the line of sight within the peripheral treatment gradient of the MYOpis Classic lens. Specifically, using the formula d = (VD + COR) × tan(θ), where vertex distance (VD) = 12 mm and the center of rotation (COR) of the eye for horizontal movements is located approximately 15 mm posterior to the corneal apex [14], a 22° gaze deviation displaces the line of sight approximately 10.91 mm from the optical center of the lens—placing it 2.91 mm beyond the 8 mm radius central viewing zone boundary and directly within the peripheral treatment gradient. To ensure head stability and maintain a fixed vertex distance throughout all measurements, participants were positioned using a standardized chin and forehead rest during all visual acuity and contrast sensitivity assessments. Off-axis gaze positions were achieved through pure ocular rotation rather than head movement, ensuring the line of sight passed consistently through the intended eccentricity of the lens at each test position. Distance visual acuity was measured at a viewing distance of 3 m using the Tomey Chart Panel TCP-3000P (Tomey, Nürnberg, Germany), while near visual acuity was evaluated using a standard Jaeger near vision card (Haag-Streit AG, Köniz, Switzerland). All VA measurements were scored by line, with a resolution of 0.1 logMAR per line. Measurements were obtained while the left eyes were occluded and participants wore each of the two spectacle lens designs under investigation.

Distance contrast sensitivity was measured monocularly (right eyes) under the same photopic conditions (426 lux) using the Tomey Chart Panel TCP-3000P at a viewing distance of 3 m. Contrast sensitivity was assessed at five spatial frequencies (1.5, 3, 6, 12, and 18 cycles per degree) and measured in the same three gaze positions (central on-axis, 22° nasal off-axis, and 22° temporal off-axis) while left eyes were occluded and participants wore each spectacle lens type. When measuring CS at each spatial frequency, three stimulus orientations were presented: vertical, 105° leftward tilt, and 75° rightward tilt. Each spatial frequency was tested at fourteen distinct contrast levels. A descending psychophysical paradigm required subjects to identify orientation, and the contrast at the last correct response established the threshold. Recorded contrast sensitivity levels were subsequently transformed into logarithms for each spatial frequency. Contrast sensitivity data were used to construct individual contrast sensitivity functions, and the area under the logarithmic contrast sensitivity function (AULCSF) was calculated using the trapezoidal integration method (AUC = ½(C_1_ + C_2_)(t_2_ − t_1_)). The order of lens conditions (SV vs. MC) was fully randomized across participants using a balanced design to control for fatigue and learning effects. The order of gaze positions (central on-axis, 22° nasal off-axis, and 22° temporal off-axis) was also counterbalanced within each lens condition to minimize order effects. Participants were allowed approximately 3 min of free viewing after donning each lens condition before formal measurements commenced. No extended wash-in or adaptation period was implemented.

### 2.4. Visual Field Sensitivity

Visual field sensitivity of right eyes was assessed using automated static perimetry (Octopus 900, Haag-Streit, AG, Köniz, Switzerland) with the TOP 30–2 protocol. This test performed twice for the right eye: once while the participant wore MC spectacles and once with SV spectacles. The order of lens conditions (SV vs. MC) for visual field testing was randomized across participants to control for learning and fatigue effects. During the test, the subject sat in front of the device, focused on a central fixation point, and pressed a button each time they detected a light stimulus presented in various locations across the visual field. All visual field tests met reliability criteria, with false positive rates, false negative rates, and fixation loss rates all below 10%.

### 2.5. Video Game Performance Test

All testing was conducted in a controlled laboratory environment with ambient illumination maintained at 300 lux. Participants completed two standardized tasks on the Aiming.Pro platform (Aim Pro Ltd., Cardiff, UK) using an HP laptop a 15.6-inch screen placed at distance of 50 cm. The tasks involved a “switch” task involving multiple targets and a “track” task involving a single moving target. Each task was performed twice, once while wearing single vision lenses and once while wearing myopia control lenses, with a randomized lens order across participants. Each participant completed both lens conditions in randomized order, with a minimum 10-min rest period between conditions to minimize fatigue and learning effects. Primary outcome measures included switching accuracy, number of switching targets destroyed, tracking accuracy, number of tracking targets destroyed, and time to task completion (seconds). Accuracy for both tasks was defined as the platform-reported hit rate, calculated as the number of successful target hits divided by total shots fired, expressed as a percentage (hits/shots × 100%).

### 2.6. Statistical Analysis

To compare the results of single vision versus myopia control lens designs repeated measures ANOVA was conducted. Data normality was assessed through visual inspection of Q-Q plots and the Shapiro-Wilk test, while the assumption of sphericity was evaluated using Mauchly’s test. In instances where sphericity was violated, degrees of freedom were adjusted using the Greenhouse-Geisser correction. Post-hoc pairwise comparisons were performed for significant main effects or interactions using the Bonferroni correction to maintain a family-wise significance level of 0.05. All statistical analyses were executed using IBM SPSS Statistics (version 29.0).

## 3. Results

The participant sample consisted of 39 individuals, characterized primarily as young adults with myopia. Demographic and ocular baseline data, including age, gender distribution, spherical equivalent, and axial length, are summarized in detail in the accompanying Table 1.

### 3.1. Impact on Visual Acuity

**Figure 2:**
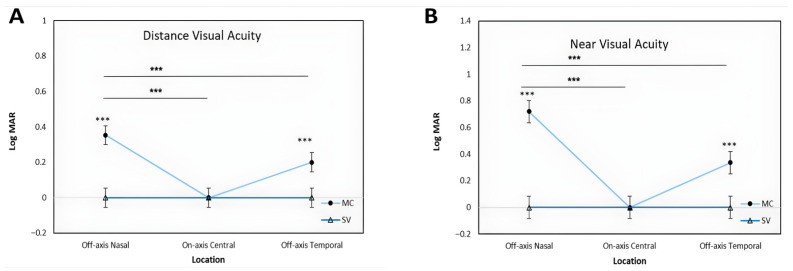
Comparison of Distance Visual Acuity (**A**) and Near Visual Acuity (**B**) between Single Vision (SV) and Myopia Control (MC) lenses across three gaze locations (Off-axis Nasal, On-axis Central, Off-axis Temporal). Visual acuity is presented in LogMAR, where lower values indicate better performance. Error bars represent 95% confidence intervals (CI), and asterisks (***) denote statistically significant differences (*p* < 0.001) between lens types at each measured location or between measured gaze positions.

For distance visual acuity (Figure 2A), SV lenses maintained excellent performance across all gaze positions, with a mean LogMAR of 0.00 ± 0.00 at all three locations (central, nasal, and temporal). MC lenses similarly preserved central distance VA (0.00 ± 0.00 LogMAR), with no significant difference from SV lenses at this position. However, MC lenses produced significant reductions in off-axis distance VA: nasal off-axis mean was 0.35 ± 0.15 LogMAR and temporal off-axis mean was 0.20 ± 0.14 LogMAR (both *p* < 0.001 vs. SV).

A similar pattern was observed for near visual acuity (Figure 2B). SV lenses demonstrated stable near-perfect acuity across all positions (0.00 ± 0.00 LogMAR centrally, nasally, and temporally). MC lenses preserved central near VA (0.00 ± 0.00 LogMAR; *p* > 0.05 vs. SV) but produced pronounced reductions in off-axis near performance: nasal off-axis near acuity was 0.72 ± 0.25 LogMAR and temporal off-axis near acuity was 0.34 ± 0.20 LogMAR (both *p* < 0.001 vs. SV). The nasal off-axis near VA reduction with MC lenses was substantially greater than the corresponding distance reduction, reflecting the additional optical burden imposed by the treatment zone during convergent near gaze.

### 3.2. Impact on Contrast Sensitivity

**Figure 3:**
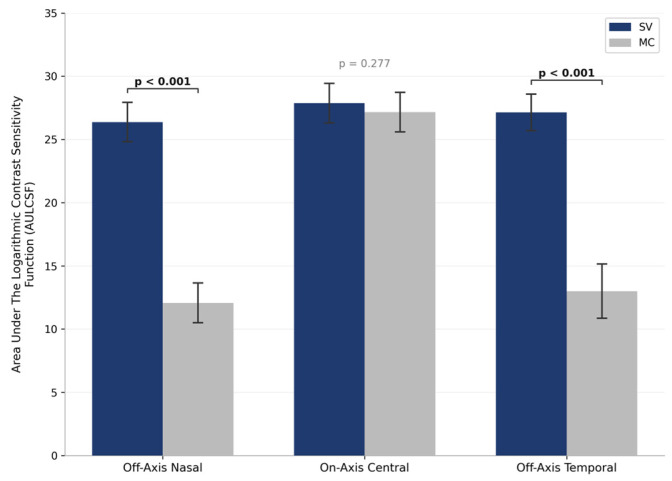
Comparison of the Area Under the Logarithmic Contrast Sensitivity Function (AULCSF) between Single Vision (SV) and Myopia Control (MC) lens designs across three gaze positions (Off-Axis Nasal, On-Axis Central, Off-Axis Temporal). Error bars represent 95% confidence interval.

The Area Under the Logarithmic Contrast Sensitivity Function (AULCSF) was compared between single vision (SV) and myopia control (MC) lens conditions across three gaze positions (Figure 3). A significant main effect of lens type was observed in the off-axis nasal position: the SV group demonstrated a substantially higher AULCSF of 26.37 ± 5.00 units compared to the MC group (12.07 ± 5.06 units; *p* < 0.001). In the on-axis central gaze position, AULCSF values were comparable between groups (SV: 27.87 ± 5.04 units; MC: 27.15 ± 5.02 units; *p* = 0.277). Consistent with the nasal findings, the off-axis temporal position also revealed a significant difference, with the SV group exhibiting a greater AULCSF (27.14 ± 4.64 units) than the MC group (13.00 ± 6.93 units; *p* < 0.001). The underlying mean ± SD log contrast sensitivity values at each spatial frequency contributing to these AULCSF calculations are provided in Supplementary Table S1, allowing independent verification of the trapezoidal integration. These results indicate that while central contrast sensitivity remains comparable between lens types, the MC lens design significantly reduces off-axis AULCSF compared to single vision correction.

**Figure 4:**
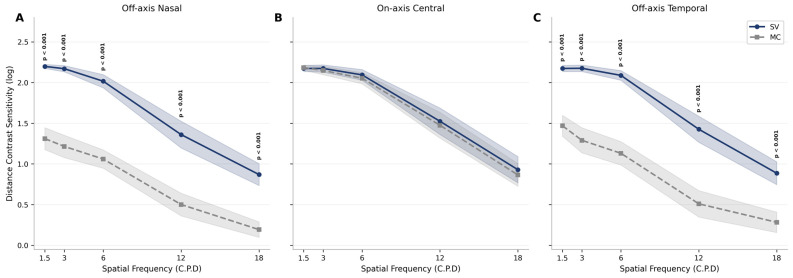
Distance contrast sensitivity across spatial frequencies (1.5 to 18 cycles per degree, C.P.D) and gaze locations ((**A**): Off-axis Nasal, (**B**): On-axis Central, (**C**): Off-axis Temporal) for single vision (SV) and myopia control (MC) lenses. Shaded areas denote 95% confidence intervals.

Additionally, distance contrast sensitivity (CS) was measured across spatial frequencies ranging from 1.5 to 18 cycles per degree (C.P.D) in three gaze positions for both single vision (SV) and myopia control (MC) lenses. In the off-axis nasal and off-axis temporal positions, the SV lenses consistently maintained significantly higher contrast sensitivity compared to the MC lenses across the entire range of spatial frequencies tested (*p* < 0.001) at all measured points. The difference between the lens types was substantial in the periphery, with the MC group showing a marked reduction in performance. In contrast, for the on-axis central gaze position, both lens types performed similarly across all spatial frequencies. Overall, these findings confirm that central contrast sensitivity remains fully preserved with MC lenses across all tested spatial frequencies (1.5–18 cpd; all *p* > 0.05), while off-axis contrast sensitivity is substantially and significantly reduced across the entire spatial frequency spectrum in both nasal and temporal gaze positions compared to SV lenses (all *p* < 0.001; Figure 4; Supplementary Table S1).

### 3.3. Impact on Visual Field Sensitivity

There were no significant differences between MC and SV lenses in Mean Deviation (MD) (*p* = 0.462) or Pattern Standard Deviation (PSD) (*p* = 0.207), suggesting that overall and localized visual field quality remained relatively stable between lens types. Averaged Visual field sensitivity of the overall tested retinal region did not differ significantly between the two lens designs (Table 2).

**Figure 5:**
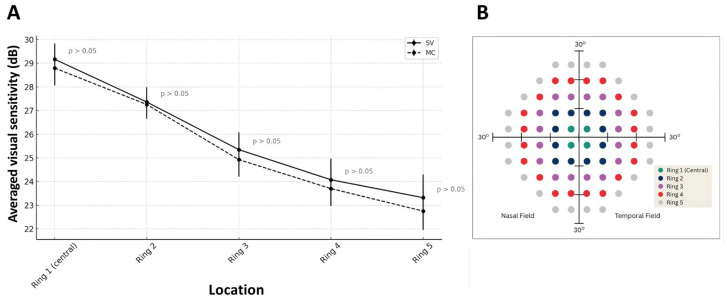
Interaction plot showing the effect of lens type (SV vs. MC) and visual field location (eccentricity) on averaged visual sensitivity (**A**), segmented numerical sensitivity plot into five concentric rings (**B**).

To investigate whether a reduction in retinal sensitivity exists toward the periphery across the measured visual field, the data from the numerical sensitivity plot were segmented into five concentric rings and subsequently averaged, as illustrated in Figure 5B. the results revealed that retinal sensitivity significantly decreased from the center to the periphery (*p* < 0.001), consistent with the known anatomical gradient. The interaction between lens type (Single Vision vs. Myopia Control) and visual field eccentricity (from central to peripheral) on average visual sensitivity was not significant (*p* = 0.937) (Figure 5A). The plot demonstrates a clear decrease in sensitivity from the center toward the periphery (*p* < 0.001), reflecting the normal anatomical gradient of the retina. Although SV lenses consistently showed higher sensitivity than MC lenses at all locations, the interaction effect was not significant indicating that the pattern of sensitivity decline across locations was similar for both lens types (Figure 5).

Spearman rank correlation analyses were performed between spherical equivalent (SE) and ring-averaged visual sensitivity across all five concentric eccentricity rings. After Bonferroni correction for five comparisons (adjusted α = 0.01), statistically significant positive correlations between SE and visual sensitivity were observed in Ring 4 (ρ = 0.42, 95% CI: 0.18–0.62, *p* = 0.007) and Ring 5 (ρ = 0.45, 95% CI: 0.21–0.64, *p* < 0.001), indicating that more myopic eyes demonstrated reduced peripheral visual sensitivity regardless of lens type. Correlations in Rings 1–3 did not survive correction (Ring 1: ρ = 0.28, *p* = 0.083; Ring 2: ρ = 0.33, *p* = 0.044; Ring 3: ρ = 0.38, *p* = 0.014).

### 3.4. Impact on Video Game Performance

No significant differences were observed between SV and MC lenses for switching accuracy (*p* = 0.331), switching targets destroyed (*p* = 0.186), tracking accuracy (*p* = 0.432), or tracking targets destroyed (*p* = 0.304). However, a significant difference was found for task completion time. The distribution of time differences was found to violate normality (Shapiro–Wilk *p* = 0.009); therefore, a Wilcoxon signed-rank test was performed. The task was completed significantly faster with SV lenses (18.39 ± 6.65 s) than with MC lenses (20.71 ± 10.08 s), W = 126, *p* = 0.012 (Table 3). Post-hoc analysis revealed a significant gender difference in gaming accuracy, with male participants outperforming female participants on both switching accuracy (10.16 ± 6.72% vs. 5.51 ± 1.67%; Mann-Whitney U, *p* < 0.001) and tracking accuracy (21.33 ± 6.40% vs. 12.65 ± 3.89%; *p* < 0.001). The gender × lens type interaction was non-significant for both switching (*p* = 0.485) and tracking accuracy (*p* = 0.978). No significant correlation was observed between spherical equivalent and gaming performance for either switching (ρ = −0.023, *p* = 0.890) or tracking accuracy (ρ = −0.008, *p* = 0.959).

## 4. Discussion

### 4.1. Visual Acuity and Contrast Sensitivity

This study provides a quantitative functional assessment of a myopia control (MC) spectacle lens design in comparison to conventional single vision (SV) correction. The principal finding is that while the MC lenses maintain central visual function comparable to SV lenses, they induce significant and clinically relevant reductions in both visual acuity (VA) and contrast sensitivity (CS) in off-axis gaze positions.

Our demographic data confirm a participant cohort typical of myopia control studies, comprising young adults with moderate myopia [8]. The key results align with the optical design principle of the MC lens, which incorporates multiple, continuous zones of relative positive power to create a myopic defocus signal in the peripheral retina [12,15]. The preserved on-axis visual performance for both distance and near VA and for central contrast sensitivity is expected and crucial for user acceptance, as central gaze is used for critical tasks such as reading, driving, and facial recognition [16]. This finding is consistent with prior investigations reporting negligible impact of similar MC designs on foveal vision [17,18].

Notably, Lam and colleagues evaluated visual function in children wearing Defocus Incorporated Multiple Segments (DIMS) lenses and reported that while central distance and near VA remained comparable to SV lenses, some participants experienced transient blurring in peripheral vision during the adaptation period [18]. The present study extends these observations by providing quantitative, task-specific measurements of off-axis visual performance in young adults, demonstrating that peripheral deficits that are persist under controlled testing conditions.

The 16 mm central viewing zone of the MYOpis Classic warrants specific discussion relative to other commercially available MC spectacle lens designs. Leading designs such as DIMS (MiYOSMART, HOYA Corporation, Tokyo, Japan) and H.A.L.T. (Stellest, Essilor International, Charenton-le-Pont, France) employ considerably smaller central zones of approximately 9 mm [12]. From a myopia control theory standpoint, a smaller central zone positions the treatment gradient closer to the visual axis, which may enhance the peripheral defocus signal and optimize axial length control efficacy. The larger 16 mm central zone of the MYOpis Classic directly explains the fully preserved central VA and AULCSF observed in this study—the line of sight in primary gaze remains entirely within the clear zone. However, it also means the treatment gradient begins farther from the visual axis compared to DIMS and H.A.L.T., which may reduce myopia control potency relative to these designs. Whether this represents a clinically meaningful difference in efficacy remains to be determined by dedicated longitudinal trials.

Beyond the difference in central zone diameter, the optical architecture of the treatment zone itself differs fundamentally between the MYOpis Classic and lenslet-based MC designs. DIMS and H.A.L.T. employ discrete lenslets embedded within a carrier of full distance correction, creating a treatment zone where focused and defocused areas are spatially interspersed. This preserves a degree of optical quality within the treatment zone, as the distance-corrected carrier between lenslets continues to contribute to retinal image formation. In contrast, the MYOpis Classic employs a continuous radially symmetric plus power gradient across the entire treatment zone with no interspersed distance-corrected carrier. When the line of sight enters this zone—as occurs at 22° off-axis gaze—it encounters an entirely continuous plus defocus field. This undiluted defocus gradient likely explains the magnitude of the off-axis VA and CS reductions observed here, and may account for why the functional deficits appear more pronounced than those reported with lenslet-based designs [19].

It is important to note that this study does not evaluate myopia control efficacy. The crossover design, adult sample, and absence of axial length outcomes preclude any conclusions regarding the MYOpis Classic’s effectiveness in slowing myopia progression. The clinical significance of the functional trade-offs documented here must therefore be considered alongside efficacy data from dedicated longitudinal studies.

The magnitude of the off-axis VA reductions observed with the MC lens warrants frank clinical discussion. For distance vision, the reduction to 0.35 ± 0.15 LogMAR nasally and 0.20 ± 0.14 LogMAR temporally represents a drop from 6/6 to approximately 6/12–6/18 Snellen equivalent in the nasal field and 6/9–6/12 temporally—losses that are clearly perceptible in everyday activities requiring peripheral awareness such as navigating crowded environments, detecting hazards while walking, or participating in dynamic sports. Similar off-axis VA reductions have been documented previously with MC spectacle lens designs incorporating peripheral defocus zones [19]. The near nasal off-axis reduction is more striking still: 0.72 ± 0.25 LogMAR corresponds to approximately 6/30–6/36 Snellen equivalent—a level that, if it were a patient’s best corrected central acuity, would constitute moderate visual impairment by WHO criteria. While this is an off-axis rather than central measurement, it has direct relevance for near tasks requiring nasal peripheral vision, including reading physical materials, food preparation, and craft work, particularly given that convergent near gaze rotates the eyes nasally and increases the likelihood of the line of sight passing through the treatment gradient. Clinicians should therefore counsel patients—particularly those engaged in visually demanding near tasks—about the potential for significant off-axis blur during convergent gaze when prescribing this lens design. It should be noted that the magnitude of these off-axis reductions should be interpreted in the context of fitting variability, as pantoscopic tilt and face form angle were visually rather than instrumentally confirmed. Small deviations in these parameters could influence the precise eccentricity at which the line of sight intersected the treatment gradient, and may have contributed to inter-individual variability in the observed functional deficits [20].

Contrast sensitivity testing typically provides insight into real-world visual performance beyond what high-contrast visual acuity can reveal [21]. The contrast sensitivity findings further elucidate the nature and extent of the functional compromise associated with MC lens designs. The significant reduction in the AULCSF in both nasal and temporal off-axis gaze positions with MC lenses indicates a broad impairment in detecting low-contrast objects across the visual field. More critically, the spatial frequency data reveal that this loss spans the entire tested frequency spectrum in off-axis gaze positions, consistent with optical defocus rather than a selective aberration. This is consistent with laboratory evidence that positive defocus degrades contrast sensitivity in a spatially graded manner, with myopes demonstrating greater sensitivity to defocus-induced CS loss than non-myopes [22]. Direct lensometer measurements across 8 lenses confirmed a mean add power of +1.97 ± 0.33 D nasally and +1.98 ± 0.38 D temporally at 10.91 mm from the optical center—well above the +0.50 D threshold documented by Radhakrishnan et al. [22] to produce significant CS reductions in myopic subjects. This finding suggests a generalized degradation of image quality, affecting the detection of large, low-contrast objects, such as a grey vehicle in overcast conditions, as well as finer peripheral details [23]. Preserving contrast sensitivity is essential for real-world visual function, postural stability, and fall prevention, even in young and otherwise healthy populations [24]. The fact that central CS remained statistically indistinguishable between lens types at all tested spatial frequencies (all *p* > 0.05; Supplementary Table S1) is reassuring from a regulatory and tolerability standpoint. However, reduced off-axis contrast sensitivity can affect real-world activities such as mobility, hazard detection, and spatial awareness [25].

An asymmetric reduction in performance—substantially greater nasally than temporally for near visual acuity—was observed and warrants further consideration. This disparity likely reflects the interaction between the spatial distribution of positive defocus zones within the lens design, ocular anatomy, and the geometry of the testing apparatus. During near work or reading, the eyes converge and rotate nasally, thereby positioning the more peripheral, power-altered zones of the spectacle lens directly within the visual axis. This optical effect has been previously reported as a possible cause of visual discomfort by some wearers of peripheral defocus lenses [26,27]. The present study provides objective, quantitative correlates to these previously subjective reports, thereby strengthening the evidence base for patient counselling and product refinement.

A clinically important question is whether the off-axis functional deficits documented here are permanent features of the optical design or whether they can be partially compensated by neurosensory adaptation over time. There is evidence that the visual system can adapt to chronic optical blur through neural recalibration. Studies using adaptive optics have demonstrated significant improvements in VA following 60 min of exposure to peripheral defocus in myopic subjects, with myopes exhibiting a greater degree of parafoveal blur adaptation than emmetropes [28]. In clinical settings, children wearing peripheral defocus MC spectacle lenses have been reported to adapt to changes in visual quality within days to weeks of initiating lens wear [29]. However, the degree to which objective, psychophysically measured off-axis VA and AULCSF improve with adaptation—as opposed to subjective comfort alone—remains poorly understood. The present study’s acute single-session design cannot address this question, and longitudinal studies tracking both objective functional outcomes and subjective visual experience across extended periods of MC lens wear are strongly needed to determine the extent and time course of any neural adaptation.

These findings have two key clinical implications. First, practitioners should counsel patients—especially children and young adults—about the trade-off between myopia control efficacy and peripheral visual performance, including adaptation periods and potential peripheral blur during off-axis gaze [30]. Second, comprehensive functional vision assessments, including peripheral VA and contrast sensitivity, should complement standard high-contrast VA measurements to fully characterize the risk-benefit profile of myopia control interventions [31]. Future research should investigate whether neuroadaptation occurs over extended wear and whether objective deficits correlate with patient-reported outcomes, visual comfort, and quality of life.

### 4.2. Visual Field Sensitivity

This study further investigated the impact of myopia control (MC) spectacle lenses on visual field sensitivity compared to single vision (SV) lenses. No significant differences were observed for overall retinal sensitivity, Mean Deviation, or Pattern Standard Deviation (all *p* > 0.05), indicating that MC lenses do not produce clinically meaningful alterations in visual field sensitivity.

A significant decline in sensitivity from center to periphery was observed (*p* < 0.001), consistent with normal retinal anatomy. The non-significant interaction between lens type and eccentricity (*p* = 0.937) confirms that the physiological sensitivity gradient is preserved with MC lenses.

These findings align with Liu and colleagues, who reported no significant difference in visual field sensitivity between DIMS and SV lenses in children across 76 locations [32]. They support that peripheral myopic defocus does not impair static perimetric sensitivity. The present study extends these observations to young adults.

Findings are also consistent with Kaymak and colleagues, who used microperimetry to examine several MC lens designs [33]. While some designs (MiYOSMART, DOT) showed measurable reductions, none produced clinically relevant loss, and all outperformed a Bangerter foil [33]. This reinforces that the minor, non-significant differences observed here are functionally inconsequential. Additionally, Desiato and colleagues reported that accommodative response and visual search performance were comparable across MC lens designs, further supporting that peripheral function for dynamic tasks remains intact [34].

The significant positive correlations between spherical equivalent and peripheral visual sensitivity observed in the outer eccentricity rings (Rings 4 and 5) are consistent with known structural changes associated with increasing axial length, including retinal stretching, photoreceptor density reduction, and choroidal thinning [35,36,37]. Correlations in the more central rings (Rings 1–3) did not survive Bonferroni correction, suggesting that the structural impact of myopia on retinal sensitivity becomes more pronounced toward the visual field periphery. This relationship was independent of lens type, confirming that myopia severity rather than optical correction drives peripheral sensitivity differences in this sample.

In summary, MC lenses do not compromise retinal sensitivity in a clinically meaningful manner. Preserved global indices and sensitivity gradient confirm that peripheral defocus zones do not adversely affect visual field integrity. While off-axis acuity and contrast may be reduced, detection of perimetric stimuli—essential for spatial orientation and hazard detection—remains intact.

### 4.3. Video Game Performance

The present study found that participants wearing myopia control (MC) lenses did not exhibit reduced switching or tracking accuracy compared to single vision (SV) lenses, but did take modestly longer to complete the aiming task. This pattern mirrors previous findings that progressive addition lens (PAL) spectacles can maintain adequate visual performance for many tasks, but may impose subtle costs on visual efficiency and comfort [38].

Specifically, research has demonstrated that PALs often have narrower intermediate and near viewing zones, along with unwanted peripheral astigmatism and distortion, which can impair tasks requiring precise optic flow or frequent gaze changes [39,40]. For instance, Selenow et al. [38] compared PALs with SV lenses in computer-based reading tasks and found that SV lenses performed significantly better in one task requiring wider visual angles and more fixational shifts, with a trend for better performance in another. The authors attributed this difference to the restricted intermediate channel of PALs, which presumably limits the clear field of view for tasks at intermediate distances [38].

Moreover, design characteristics and individual fitting parameters influence overall optical performance. Sheedy et al. [39] measured the optical properties of 23 different PAL designs and reported significant differences in viewing zone sizes, unwanted astigmatism, and minimum fitting heights. These differences can be used to select a PAL design matching the patient’s specific visual needs, suggesting that the modest delay in task completion observed in our study may stem not only from the inherent geometry of PALs (i.e., narrower clear-vision corridors), but also from the specific design characteristics of the lenses evaluated [39]. Additionally, a recent theoretical analysis by Pascual et al. [20] demonstrated that even small deviations in fitting parameters (i.e., vertex distance, pantoscopic tilt) can degrade the optical quality of PALs compared to optimally fitted lenses, particularly under dynamic vision conditions. This implies that individual differences in lens fitting may have contributed to the modest delay observed in our study.

Despite slower completion times, the lack of accuracy loss suggests that participants likely compensated by slowing their movements or adjusting their gaze or head position to preserve performance. Such compensation strategies have been documented among PAL wearers during sustained digital device use or visually demanding tasks [20,40]. De Lestrange-Anginieur and Kee [40] noted that the optical performance of PALs can vary significantly with astigmatic prescription and lens design, which may necessitate adaptive gaze strategies to maintain task accuracy.

The overall accuracy values of 7.25 ± 4.76% for switching and 15.68 ± 6.11% for tracking represent genuine platform-reported hit rate percentages (hits/shots × 100%). Post-hoc analysis revealed a significant gender difference in accuracy, with male participants outperforming female participants on both switching (10.16 ± 6.72% vs. 5.51 ± 1.67%; *p* < 0.001) and tracking accuracy (21.33 ± 6.40% vs. 12.65 ± 3.89%; *p* < 0.001). The gender × lens type interaction was non-significant for both tasks (switching: *p* = 0.485; tracking: *p* = 0.978), and no significant correlation was found between spherical equivalent and gaming performance (switching: ρ = −0.023, *p* = 0.890; tracking: ρ = −0.008, *p* = 0.959), confirming that neither gender nor refractive error confounded the between-lens comparisons. The low accuracy values reflect the high task difficulty for non-gaming participants and the predominantly female sample composition. It should be noted that these values indicate a floor effect, with performance near the lower boundary of the dynamic range of the hit rate metric for non-gaming participants. This may limit the sensitivity of accuracy as a measure for detecting subtle lens-induced differences. The completion time outcome, which was not subject to the same floor constraint, may therefore represent a more sensitive performance indicator in this population.

In summary, our findings are consistent with literature indicating that MC lenses do not necessarily degrade visuomotor precision, but may impose a small cost in speed, especially in tasks requiring rapid, dynamic visual processing. Future investigations should assess whether these effects are exacerbated in populations less well-adapted to PALs (i.e., older adults or first-time wearers), and whether optimization of lens design or fitting parameters can mitigate the observed delay.

### 4.4. Limitations

Several limitations should be acknowledged. First, the sample comprised only young adult myopes, so findings may not generalize to children with greater neural plasticity [18] or to older adults with different adaptive strategies [38]. Second, testing used static gaze positions; dynamic tasks involving coordinated eye-head movements may reveal different functional impacts [20,38], as suggested by the modest delay in video game completion with MC lenses. Third, only one MC lens design was evaluated, and results may not generalize to all commercially available designs given variability in optical design parameters [20,40]. Fourth, while vertex distance was measured for each participant and maintained at 12 mm, pantoscopic tilt and face form angle were visually confirmed by the examiner during frame positioning rather than instrumentally measured. This introduces a degree of subjectivity in these fitting parameters that may have contributed to inter-individual variability in the precise eccentricity at which the line of sight intersected the lens treatment gradient during off-axis testing. However, given that the line of sight at 22° passes through the lens at approximately 10.91 mm from the optical center—2.91 mm beyond the central zone boundary—this degree of fitting variability is unlikely to have materially affected whether participants were viewing through the treatment gradient during off-axis measurements. Future studies should consider instrumental measurement of all fitting parameters to improve optical geometry standardization. In particular, the magnitude of the off-axis VA and CS reductions reported here should be interpreted with the caveat that pantoscopic tilt and face form angle were visually rather than instrumentally confirmed, and individual variability in these parameters may have contributed to the observed functional deficits. Fifth, the correlation between spherical equivalent and peripheral visual field sensitivity highlights that more myopic eyes inherently have reduced sensitivity due to anatomical changes, which may have masked small lens-induced differences.

Sixth, all measurements were performed under acute conditions without a structured adaptation. Participants wore each lens for only the duration of the testing session, which does not reflect the visual experience of habitual long-term wearers. It is well established that the visual system can partially adapt to chronic optical blur through neural recalibration of spatial contrast processing [27,30]. Habitual wearers of MC lenses may therefore experience reduced subjective awareness of peripheral blur over weeks to months of continuous wear, even if the objective optical defocus remains unchanged. The functional deficits quantified in this study—particularly the off-axis VA and CS reductions—may consequently overestimate the perceptual impact experienced in daily life by adapted wearers. Longitudinal studies tracking both objective functional outcomes and subjective visual experience across extended periods of MC lens wear are needed to determine the extent and time course of any neural adaptation.

Finally, the absence of patient-reported outcome measures represents a notable gap in the current evidence. Objective functional measurements, as reported here, do not necessarily translate directly into patient-perceived visual disability or discomfort in daily life. Validated instruments such as the National Eye Institute Visual Function Questionnaire (NEI-VFQ-25) or MC lens-specific comfort and adaptation questionnaires [13,18] would provide complementary insight into how the functional trade-offs documented here are experienced by wearers. Subjective visual comfort, perceived blur, and adaptation are important determinants of long-term compliance with MC lens wear, and their absence from the present study limits the translational completeness of our findings. Future research should integrate patient-reported outcome measures alongside objective visual function assessments to provide a more complete characterization of the real-world impact of MC spectacle lens designs.

It is important to note that this study does not evaluate myopia control efficacy. The single-session, within-subject crossover design, adult sample, and absence of axial length outcomes preclude any conclusions regarding the MYOpis Classic’s effectiveness in slowing myopia progression. The clinical significance of the functional trade-offs documented here must therefore be considered alongside efficacy data from dedicated longitudinal trials.

## 5. Conclusions

This study demonstrates that myopia control lenses with concentric peripheral defocus zones preserve central visual function—including on-axis acuity, central contrast sensitivity, and visual field indices—while inducing significant off-axis reductions in acuity and contrast sensitivity. Peripheral deficits were more pronounced nasally and for near tasks, consistent with the intended myopic defocus design. Retinal sensitivity remained unaffected, and dynamic accuracy was maintained; however, task completion speed was modestly reduced, suggesting compensatory strategies. The correlation between spherical equivalent and peripheral sensitivity highlights the importance of considering baseline refractive error. It should be noted that this study evaluates functional trade-offs only and does not provide evidence of myopia control efficacy for this specific lens design. The clinical significance of the functional costs documented here must be weighed against efficacy data from dedicated longitudinal studies.

Clinically, practitioners should counsel patients on functional trade-offs, particularly for those engaged in visually demanding activities, and anticipate an adaptation period with possible peripheral blur. Longitudinal and comparative research across lens designs is needed. This work provides an evidence base for informed decision-making, reinforcing that myopia control involves a compromise between long-term ocular benefits and immediate visual function.

## Figures and Tables

**Table 1:** Participant Baseline Demographic and Ocular Characteristics.

Characteristic	Value	Range
Sample size (n)	39	
Age (years)	21.1 ± 1.3	19–24
Spherical Equivalent (D)	−2.85 ± 2.84	−10.0–0.0
Axial Length (mm)	24.35 ± 1.56	22.50–28.10
Gender	Female: 24, Male: 15	

**Table 2:** Visual field performance metrics with single vision and myopia control lens groups.

	Single Vision Lens (Mean ± SD)	Myopia Control Lens (Mean ± SD)	*p*-Value
Mean Deviation decibels (dB)	−2.7 ± 1.8	−2.9 ± 1.6	0.462
Pattern Standard Deviation decibels (dB)	2.45 ± 1.3	2.86 ± 0.9	0.207
Overall averaged retinal sensitivity decibels (dB)	25.85 ± 3.2	25.49 ± 3.12	0.11

**Table 3:** Comparison of performance metrics between myopia control (MC) and single vision (SV) lenses. Data are presented as mean ± standard deviation (SD). The asterisk (*) denoting a significant difference.

Task	MC (Mean ± SD)	SV (Mean ± SD)	*p*-Value
Switching Accuracy (%) ^†^	7.25 ± 4.76	7.22 ± 4.87	0.331
Switching Targets Destroyed (count)	64.10 ± 31.28	68.85 ± 36.71	0.186
Tracking Accuracy (%) ^†^	15.68 ± 6.11	15.99 ± 6.83	0.432
Tracking Targets Destroyed (count)	168.73 ± 75.46	175.78 ± 82.24	0.304
Completion Time (seconds)	20.71 ± 10.08	18.39 ± 6.65	0.012 *

^†^ Accuracy represents the platform-reported hit rate (targets successfully hit/total shots fired × 100%). * Statistically significant (Wilcoxon signed-rank test; Shapiro-Wilk). All other comparisons by Wilcoxon signed-rank test.

## Data Availability

The data presented in this study are available on request from the corresponding author.

## Supplementary Materials

The following supporting information can be downloaded at: https://www.mdpi.com/article/10.3390/jcm15093362/s1. Table S1: Mean ± SD log contrast sensitivity values at each spatial frequency, gaze position, and lens type. Figure S1: Spearman rank correlations between spherical equivalent (SE) and AULCSF across three gaze positions (central, nasal off-axis, and temporal off-axis) for SV and MC lens conditions (n = 39). Each panel shows individual data points with a fitted regression line. Correlation coefficients (ρ) and *p*-values are displayed within each panel.

## Abbreviations

The following abbreviations are used in this manuscript:

MC	Myopia control
SV	Single vision
CPD	Cycles per degree

## References

[1] Flitcroft D.I. (2012). The complex interactions of retinal, optical and environmental factors in myopia aetiology. Prog. Retin. Eye Res..

[2] Holden B.A., Fricke T.R., Wilson D.A., Jong M., Naidoo K.S., Sankaridurg P., Wong T.Y., Naduvilath T.J., Resnikoff S. (2016). Global prevalence of myopia and high myopia and temporal trends from 2000 through 2050. Ophthalmology.

[3] Morgan I.G., French A.N., Ashby R.S., Guo X., Ding X., He M., Rose K.A. (2018). The epidemics of myopia: Aetiology and prevention. Prog. Retin. Eye Res..

[4] Wong T.Y., Ferreira A., Hughes R., Carter G., Mitchell P. (2014). Epidemiology and disease burden of pathologic myopia and myopic choroidal neovascularization: An evidence-based systematic review. Am. J. Ophthalmol..

[5] Haarman A.E.G., Enthoven C.A., Tideman J.W.L., Tedja M.S., Verhoeven V.J.M., Klaver C.C.W. (2020). The complications of myopia: A review and meta-analysis. Investig. Ophthalmol. Vis. Sci..

[6] Tideman J.W.L., Snabel M.C.C., Tedja M.S., van Rijn G.A., Wong K.T., Kuijpers R.W.A.M., Vingerling J.R., Hofman A., Buitendijk G.H.S., Keunen J.E.E. (2016). Association of axial length with risk of uncorrectable visual impairment for Europeans with myopia. JAMA Ophthalmol..

[7] Smith E.L., Hung L.F., Huang J. (2009). Relative peripheral hyperopic defocus alters central refractive development in infant monkeys. Vis. Res..

[8] Sankaridurg P., Donovan L., Varnas S., Ho A., Chen X., Martinez A., Fisher S., Lin Z., Smith E.L., Ge J. (2010). Spectacle lenses designed to reduce progression of myopia: 12-month results. Optom. Vis. Sci..

[9] Chakraborty R., Ostrin L.A., Nickla D.L., Iuvone P.M., Pardue M.T., Stone R.A. (2018). Circadian rhythms, refractive development, and myopia. Ophthalmic Physiol. Opt..

[10] Chia A., Lu Q.S., Tan D. (2016). Five-year clinical trial on atropine for the treatment of myopia 2: Myopia control with atropine 0.01% eyedrops. Ophthalmology.

[11] Walline J.J., Lindsley K.B., Vedula S.S., Cotter S.A., Mutti D.O., Ng S.M., Twelker J.D. (2020). Interventions to slow progression of myopia in children. Cochrane Database Syst. Rev..

[12] Lam C.S.Y., Tang W.C., Tse D.Y., Lee R.P.K., Chun R.K.M., Hasegawa K., Qi H., Hatanaka T., To C.H. (2020). Defocus Incorporated Multiple Segments (DIMS) spectacle lenses slow myopia progression: A 2-year randomised clinical trial. Br. J. Ophthalmol..

[13] Lipson M.J., Boland B., McAlinden C. (2022). Vision-related quality of life with myopia management: A review. Cont. Lens Anterior Eye.

[14] Ohlendorf A., Schaeffel F., Wahl S. (2022). Positions of the horizontal and vertical centre of rotation in eyes with different refractive errors. Ophthalmic Physiol. Opt..

[15] Applegate R.A., Ballentine C., Gross H., Sarver E.J., Sarver C.A. (2003). Visual acuity as a function of Zernike mode and level of root mean square error. Optom. Vis. Sci..

[16] Bao J., Huang Y., Li X., Yang A., Zhou F., Wu J., Wang C., Li Y., Lim E.W., Spiegel D.P. (2022). Spectacle lenses with aspherical lenslets for myopia control vs single-vision spectacle lenses: A randomized clinical trial. JAMA Ophthalmol..

[17] Gao Y., Lim E.W., Yang A., Drobe B., Bullimore M.A., Chen X. (2021). The impact of spectacle lenses for myopia control on visual functions. Ophthalmic Physiol. Opt..

[18] Lam C.S.Y., Tang W.C., Qi H., Radhakrishnan H., Hasegawa K., To C.H., Charman W.N. (2020). Effect of defocus incorporated multiple segments spectacle lens wear on visual function in myopic Chinese children. Transl. Vis. Sci. Technol..

[19] Gao Y., Lim E.W., Drobe B. (2023). Impact of myopia control spectacle lenses with highly aspherical lenslets on peripheral visual acuity and central visual acuity with peripheral gaze. Ophthalmic Physiol. Opt..

[20] Pascual E., González C., Alonso J., Gómez-Pedrero J.A. (2023). Theoretical performance of progressive addition lenses with poorly measured individual parameters. Ophthalmic Physiol. Opt..

[21] Owsley C. (2003). Contrast sensitivity. Ophthalmol. Clin. N. Am..

[22] Radhakrishnan H., Pardhan S., Calver R.I., O’Leary D.J. (2004). Effect of positive and negative defocus on contrast sensitivity in myopes and non-myopes. Vis. Res..

[23] Lord S.R., Menz H.B. (2000). Visual contributions to postural stability in older adults. Gerontology.

[24] Wood J.M., Lacherez P.F., Anstey K.J. (2013). The on-road difficulties of older drivers and their relationship with self-reported motor vehicle crashes. J. Am. Geriatr. Soc..

[25] West S.K., Rubin G.S., Broman A.T., Muñoz B., Bandeen-Roche K., Turano K. (2002). How does visual impairment affect performance on tasks of everyday life? The SEE Project. Arch. Ophthalmol..

[26] Shah S., Motwani G., Verkicharla P.K. (2025). “REACH” for troubleshooting peripheral defocus myopia control spectacles. Front. Ophthalmol..

[27] Lu Y., Lin Z., Wen L., Gao W., Pan L., Li X., Yang Z., Lan W. (2020). The adaptation and acceptance of defocus incorporated multiple segment lens for Chinese children. Am. J. Ophthalmol..

[28] Ghosh A., Zheleznyak L., Barbot A., Jung H., Yoon G. (2017). Neural adaptation to peripheral blur in myopes and emmetropes. Vis. Res..

[29] Fatimah M., Agarkar S., Narayanan A. (2024). Impact of defocus incorporated multiple segments (DIMS) spectacle lenses for myopia control on quality of life of the children: A qualitative study. BMJ Open Ophthalmol..

[30] Wildsoet C.F., Chia A., Cho P., Guggenheim J.A., Polling J.R., Read S., Sankaridurg P., Saw S.-M., Trier K., Walline J.J. (2019). IMI interventions myopia institute: Interventions for controlling myopia onset and progression report. Investig. Ophthalmol. Vis. Sci..

[31] Janarthanan S.D., Samiyullah K., Madheswaran G., Ballae Ganeshrao S., Watt K. (2024). Exploring the impact of optical corrections on visual functions in myopia control—A scoping review. Int. Ophthalmol..

[32] Liu K.K.K., Zhang H.Y., Leung K.Y., Tse D.Y.Y., Lam C.S.Y. (2024). Evaluation of the visual field performance of Defocus Incorporated Multiple Segments lenses (DIMS) versus single vision lenses. Investig. Ophthalmol. Vis. Sci..

[33] Kaymak H., Mattern A.I., Graff B., Devenijn M., Seitz B., Schwahn H. (2025). Optical influence of myopia control spectacles at the retinal level: Effect of local light modulation. Ophthalmic Physiol. Opt..

[34] Desiato A., Lam H.Y., Rani Anand R., Chatha I., Logan N.S., Sheppard A., Wolffsohn J.S., Laughton D., Davies L.N. (2025). The impact of myopia control spectacle lens designs on visual function. Ophthalmic Physiol. Opt..

[35] Jonas J.B., Spaide R.F., Ostrin L.A., Logan N.S., Flitcroft I., Panda-Jonas S. (2023). IMI-Nonpathological Human Ocular Tissue Changes with Axial Myopia. Investig. Ophthalmol. Vis. Sci..

[36] Wang Y., Ye J., Shen M., Yao A., Xue A., Fan Y., Huang S., Wang J., Lu F., Shao Y. (2019). Photoreceptor degeneration is correlated with the deterioration of macular retinal sensitivity in high myopia. Investig. Ophthalmol. Vis. Sci..

[37] Alanazi M., Caroline P., Alshamrani A., Alanazi T., Liu M. (2021). Regional distribution of choroidal thickness and diurnal variation in choroidal thickness and axial length in young adults. Clin. Ophthalmol..

[38] Selenow A., Bauer E.A., Ali S.R., Spencer L.W., Ciuffreda K.J. (2002). Assessing visual performance with progressive addition lenses. Optom. Vis. Sci..

[39] Sheedy J., Hardy R.F., Hayes J.R. (2006). Progressive addition lenses—Measurements and ratings. Optometry.

[40] De Lestrange-Anginieur E., Kee C.S. (2021). Optical performance of progressive addition lenses (PALs) with astigmatic prescription. Sci. Rep..

