# Supplementary material for: Impact of Myopia Control Spectacle Lenses on Visual Functions in Young Adults: A Comprehensive Evaluation
Source: J Clin Med. 2026 Apr 28;15(9):3362. doi: 10.3390/jcm15093362 (PMC13163369; doi:10.3390/jcm15093362)
Supplement: Supplementary file 1 [file jcm-15-03362-s001.zip › jcm-4246264-supplementary.pdf]

Journal of
Clinical Medicine

MDPI

Table S1. Mean ± SD Log Contrast Sensitivity at Each Spatial Frequency, Gaze Position, and Lens

Gaze Position	Spatial Frequency (C.P.D)	Single Vision (SV)	Myopia Control (MC)	Comparison (SV vs MC)
		Mean Log CS ± SD	Mean Log CS ± SD	p-value
Off-Axis Nasal	1.5	2.198 ± 0.079	1.311 ± 0.428	< 0.001
	3	2.170 ± 0.122	1.215 ± 0.437	< 0.001
	6	2.016 ± 0.259	1.060 ± 0.359	< 0.001
	12	1.360 ± 0.523	0.501 ± 0.444	< 0.001
	18	0.869 ± 0.427	0.193 ± 0.302	< 0.001
On-Axis Central	1.5	2.173 ± 0.121	2.184 ± 0.098	0.570
	3	2.173 ± 0.141	2.148 ± 0.164	0.463
	6	2.094 ± 0.208	2.053 ± 0.217	0.357
	12	1.524 ± 0.519	1.475 ± 0.505	0.594
	18	0.929 ± 0.514	0.865 ± 0.444	0.438
Off-Axis Temporal	1.5	2.173 ± 0.121	1.469 ± 0.409	< 0.001
	3	2.175 ± 0.127	1.290 ± 0.492	< 0.001
	6	2.088 ± 0.189	1.131 ± 0.457	< 0.001
	12	1.426 ± 0.510	0.510 ± 0.518	< 0.001
	18	0.886 ± 0.447	0.283 ± 0.400	< 0.001

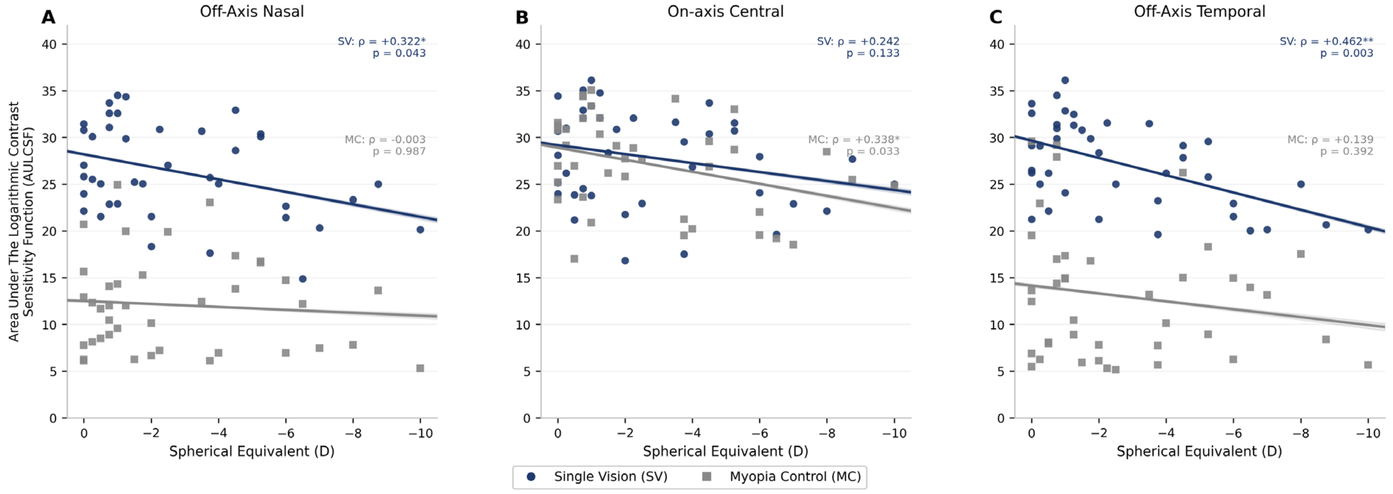

Figure S1. Spearman rank correlations between spherical equivalent (SE) and AULCSF across three gaze positions (central, nasal off-axis, and temporal off-axis) for SV and MC lens conditions (n = 39). Each panel shows individual data points with a fitted regression line. Correlation coefficients (ϱ) and p-values are displayed within each panel.

https://doi.org/10.3390/xxxxx
